# The Relationship Between Children and Their Maternal Uncles: A Unique Parenting Mode in Mosuo Culture

**DOI:** 10.3389/fpsyg.2022.873137

**Published:** 2022-05-20

**Authors:** Erping Xiao, Jing Jin, Ze Hong, Jijia Zhang

**Affiliations:** ^1^Jing Hengyi School of Education, Hangzhou Normal University, Hangzhou, China; ^2^Foreign Languages Teaching Center, Shanghai University of Traditional Chinese Medicine, Shanghai, China; ^3^Department of Human Evolutionary Biology, Harvard University, Cambridge, MA, United States; ^4^Department of Sociology, Zhejiang University, Hangzhou, China; ^5^Faculty of Education, Guangxi Normal University, Guilin, China

**Keywords:** attachment, parent–child relation, maternal uncle–child relation, the Mosuo, the Han

## Abstract

The relationship between children and their maternal uncles in contemporary Mosuo culture reveals a unique parenting mode in a matrilineal society. This study compared the responses of Mosuo and Han participants from questionnaires on the parent–child and maternal uncle–child relationship. More specifically, Study 1 used Inventory of Parent and Peer Attachment (IPPA) to assess the reactions of the two groups to the relationship between children and their mothers, fathers, and maternal uncles. The results show that while Han people display a higher level of attachment toward their fathers than their maternal uncles, Mosuo people do not exhibit a significant difference in this aspect. Study 2 used a scenario-based method to compare how adults and teenagers perceive the rights and responsibilities of fathers/maternal uncles toward their children/nephews or nieces. The results show that Han adults attribute more rights and responsibilities to their own children than nephews/nieces, while their Mosuo counterparts have the reverse pattern and assign stronger responsibilities to their nephews/nieces than their own children. Both groups perceive the fathers to be the bearer of rights and responsibilities, although this perception was weaker among Mosuo. This paper concludes that in the Mosuo society, fathers have a relatively weak social role as a result of their unique matrilineal social structure.

## Introduction

The Mosuo (also known as the Na) is an ethnic minority group inhabiting the shores of Lugu Lake in southwest China, which has common borders with Yunnan Province and Sichuan Province. Traditionally, Mosuo’s subsistence system mainly consisted of hunting, fishing, and small-scale farming (Cai, 2002), though in recent decades the Mosuo society has also been influenced by commercialization and tourism. Ethnographically, the Mosuo is probably most famous for its unique marriage pattern, the walking marriage where either woman or man will marry the other freely. The lovers meet at woman’s house at night and the man returns to his own maternal family at dawn (Mathieu, 2000; Walsh, 2005), making it one of the very few societies where neither the males nor the females disperse after marriage (known as duolocal residence; Ji et al., 2013). Ethnographic descriptions suggest that walking marriages involve no formal contract between lovers (Shih, 2008) with the indication that paternity is often not assured and relatively unimportant among the Mosuo, and multiple concurrent romantic unions are possible and do not invoke much jealously (Cai, 2002).

In a traditional Mosuo family, women are perceived to carry the blood relation among relatives and take on many important household responsibilities in the household, including organizing agricultural production, raising children, and arranging daily chores (Zhou, 2010). As the lovers will not set up a new family and do not share property. Any children resulting from the union of the lovers are the woman’s children and the man helps to raise the children of his sisters. Children live with their mothers, maternal uncles, grandmothers, and other maternal family members, and do not interact much with their biological fathers (Zhan et al., 2006). In line with this, men in matrilineal societies not only serve as the role of father and husband but also very importantly maternal uncle, brother, and son (Ji et al., 2013; Wu et al., 2013). Most notably, Mosuo women’s brothers share the common blood tie with them and are perceived to belong to the same family, minimizing potential conflict of interest (e.g., distribution and inheritance of property). As a result, Mosuo men are often actively involved in assisting to raise their nephews and nieces. It is also worth noting that in traditional matrilineal societies, the dominance of father or husband in the household is often absent in contrast with patrilineal society (Xiao et al., 2015). Mosuo culture respects and emphasizes the importance of women yet at the same time does not suppresses their male counterparts, reaching a complementary harmony between males and females (Zhou, 2010).

From childhood to adulthood, individuals have different attachment styles affected by one’s early personal experiences in the environment where they grow up (Picardi et al., 2013). According to theory of attachment of Bowlby (1969), a growing individual develops an attachment to the person who takes care of him/her, especially his/her mother, and such attachment is crucial for the children’s cognitive and emotional development. Although the normative aspect and universal claims of attachment theory have been under some debate (Röttger-Rössler, 2014; Keller, 2018), there are good reasons to postulate that the quality and the extent to which children interact with their caregivers (often family members) will have some effect on their psychology as they grow up. Traditionally, attachment theory centers around the interaction between an infant/child and her caregiver as well as the cognitive and developmental consequences of such interaction or lack thereof (Schore and Schore, 2008), and cross-cultural studies usually focus on the relevance and applicability of attachment theory in non-western societies (Röttger-Rössler, 2014). In comparison, relatively little attention has been paid to the underlying social and family structures that produce various styles of attachment. This paper therefore examines a particular socio-cultural context—the matrilineal Mosuo society in southwest China—to examine how family structure affects individuals’ attachment to various family members. In addition to biological parents, special attention is paid to the child’s maternal uncle whose role is often marginalized in patrilineal societies. Through a combination of psychometric tests and vignette scenarios, this paper aims to show that the kind of attachment children form with their relatives and the actions they would make in various real-life scenarios crucially depends on the cultural context, and a full understanding of the relationships among family members likely require knowledge of the social and family structure.

Malinowski (2005) was among the first anthropologists to point out the importance of men as maternal uncles in matrilineal societies, and subsequent anthropological research has shown that men in matrilineal societies often face the decision of splitting their investment between their own children and the sisters’ children, creating a potential conflict (Richards, 2015). Although the extent to which men are involved in raising their sisters’ offspring vary across matrilineal societies, men as maternal uncles usually exert a lot of influence in the family (Starkweather and Keith, 2019). Among the Mosuo, men are well respected by their nephew/niece and enjoys high status within the household. They are often the main source of financial income of the family and are in charge of important family events, such as building a new house or planning a funeral (Zhong, 2011). In many ways, the role Mosuo men play as maternal uncles resembles that of fathers/husbands in a typical patrilineal society.

What is perhaps surprising is that the importance of men as maternal uncles was very resilient to external pressures. From the 1950s to the 1970s, The Chinese government implemented a series action to force Mosuo people to adopt monogamy, yet according to Cai (2002), none succeeded and Mosuo men continued to affiliate strongly with their sisters’ family (and from their perspective, their own family). For the last decade, policy, education, modernization, and tourism, as external driving forces, have brought about major changes within the Mosuo culture, including family size, family structure, life style, dwellings, and so on (Feng and Xiao, 2021). In contemporary Mosuo societies, with the expansion of formal schooling and the ever-present mainstream Han culture, men’s role as maternal uncles is still very prominent in comparison with nearby ethnic groups (He, 2000), though it is possible that the younger Mosuo generation is more culturally similar to the Han.

In Mosuo society, the relationship between the child and his/her maternal uncle (also known as avunculate in anthropology) is very close. The maternal uncle takes up many roles in bringing up sister’s child, including passing on important life skills and educating the child in everyday circumstances (Zhou, 2010). For the children, their maternal uncle represents the closest and most authoritative elderly man, sometimes more than their own biological father. As such, the closeness of maternal uncle–nephew relationship and the relative alienation of father–child relationship are in sharp contrast, highlighting the uniqueness of parenting mode in Mosuo culture (Peng, 1997; He, 2000). However, the current commodity economy and mainstream Han culture also has impact on Mosuo society in significant ways, thus greatly influencing Mosuo’s traditional life mode, family structure, and responsibility assignment. Under such influence, the relationship between husband and wife becomes closer and fathers start to take on more economical responsibility and the upbringing of their own children. These ongoing social changes inevitably affect the traditional Mosuo parenting mode.

Two studies were conducted to compare the Mosuo and the Han from the perspective of attachment and obligation, respectively. The present study mainly aims to answer two questions: (1) whether the pattern of parent–child attachment is affected by culture. Specifically, compared with the mainstream Han culture, whether there are differences in parent–child attachment in the Mosuo culture; (2) whether the pattern of parent–child obligation relation at different ages is affected by culture. The younger generation is supposed to be exposed more to the patrilineal Han culture than the older generation, so, whether there are generation gaps regarding the attitudes on the obligation relation. An extensive interpretation of the results in light of the specific cultural differences between the Mosuo and the Han is discussed.

## Study 1

As children’s experiences of living with their family members could influence their attachment (Picardi et al., 2013; Röttger-Rössler, 2014), three styles of attachment were compared between the Mosuo and the Han in Study 1, namely, mother attachment, father attachment, and maternal uncle attachment. In the Mosuo families, mother and maternal uncle are co-residents of children, while in the Han families, mother and father are the co-residents. In any culture, mother attachment is always considered as the most important parent–child attachment relationship, which will be no exception in Mosuo and Han culture. The attachment styles were hypothesized to reflect the traditional Mosuo family structure and culture, highlighting the closeness of uncle–nephew/niece relationship. At the same time, it may also be influenced by the contemporary social changes. For the Han participants, the attachment mode highlights the strong attachment with the father and mother, but not with maternal uncle.

### Participants

The sample consisted of 317 Mosuo people (160 males and 157 females, mean age = 16.5 ± 5.96 years) and 196 Han people (103 males and 93 females, mean ages = 15.16 ± 5.29 years). All participants are from the same local middle school and are proficient in Mandarin, ensuring the understanding of questionnaires written in Mandarin Chinese characters. The basic demographic variables including age, ethnicity, and gender were collected. The study was approved by the Scientific Research Ethics Committee of Hangzhou Normal University. Parents or legal guardians of the minors were informed of the nature and content of the study and gave either oral or written consent to allow their minor children to participate, and all the minors also gave their oral assent prior to participating in this study. All participants received a gift for completing the study.

### Materials and Procedure

This paper modified the self-reported questionnaire for an evaluation of individuals’ early attachment to parents and partners according to the Inventory of Parent and Peer Attachment (IPPA). IPPA was designed for adolescents from 12 to 19 years old, and it evaluates the perception of the quality of parent–child relationships as well as the relationships between adolescents and their close friends (Armsden and Greenberg, 1987). The theory behind IPPA focuses on attachment target and trusting relation—the cognitive dimension (Bowlby, 1973). The questionnaire is divided into three sections (Mother, Father, and Peer sections) and three dimensions are assessed, that is, trust, communication, and alienation. Trust measures the level of mutual understanding and respect, communication refers to the common contents and quality of the interaction between an individual and his/her attaching target, and alienation refers to the state of being angry with the attachment subject and emotionally alienated, which manifests as anger, interpersonal isolation, and unsafe attachment (Armsden and Greenberg, 1987).

Given the purpose of this study, the Chinese version of IPPA translated and published by Bao and Xu (2006) was used and a maternal uncle attachment subscale was added. Each subscale has 25 items and each item is rated by means of a five-point Likert scale. Scores were given for levels of disagreement and agreement (1–5), where 1 means almost never or never true, 2 means not very often true, 3 means sometimes true, 4 means often true, and 5 means almost always or always true. Trust and communication questions were scored positively and alienation questions were scored reversely. The higher the total score, the higher the perceived attachment security. In Chinese edition of IPPA (Bao and Xu, 2006), an adequate internal consistency (*α* ≥ 0.90) for each of the subscale was obtained.

Participants filled in the Chinese IPPA in the classrooms anonymously. No discussion was allowed to ensure independence of responses.

### Results

#### Reliability Analysis

To examine the reliability of the questionnaire, the internal consistency and stability was checked. The results are shown in Table 1, where the internal consistency of the three questionnaires reaches above 0.75, suggesting that all the three subscales measured the same traits. Cronbach’s alpha coefficients of the three factors in each subscale were higher than 0.60 and the reliability was medium.

**Table 1:** Reliability analysis of subscales of IPPA in Study 1.

Subscale	Cronbach’s Alpha Coefficient (*α*)	Factors		
		Trust	Communication	Alienation
Mother attachment	0.79	0.65	0.72	0.68
Father attachment	0.80	0.75	0.74	0.70
Maternal uncle attachment	0.78	0.71	0.77	0.62

#### Ethnic Differences in Attachment Types

Attachment types were designed using the IPPA classification rules. Score distributions were divided into lowest, middle, and highest third, and a rating of “low,” “medium,” or “high” was assigned accordingly. Scores falling at the cut points were designed medium security to create maximally discriminating low security and high security (Armsden and Greenberg, 1987; Vivona, 2000). Attachment types of the two ethnic groups are summarized in Table 2.

**Table 2:** Ethnic differences in attachment types in Study 1.

Subscale	Attachment types	Mosuo Group (N =317)	Han Group (N =196)	*χ* ^2^
Mother attachment	High security	23%	35%	16.80^***^
	Medium security	50%	51%
	Low security	27%	14%
Father attachment	High security	23%	38%	27.98^***^
	Medium security	47%	51%
	Low security	30%	11%
Maternal uncle attachment	High security	20%	25%	2.10
	Medium security	60%	55%
	Low security	20%	21%

The numbers in the table are the percentages of each attachment types, which are obtained by dividing the numbers of people with each attachment types by the total numbers of each ethnic.

*** *p* < 0.001.

*χ*^2^ was used to test mother attachment with participants from the two different ethnicities. The result showed significant differences in mother attachment types for different ethnic types. *χ*^2^ = 16.80, *p* < 0.001. Regarding high security attachment type, Han participants have higher proportion (35.18%) than Mosuo counterparts (22.71%). In contrast, for low security attachment type, Mosuo group shows a bigger percentage (26.82%) over Han group (13.56%). Likewise, type of attachment to father was tested by *χ*^2^. Again, significant differences were observed in type of father attachment for different ethnic backgrounds. *χ*^2^ = 27.98, *p* < 0.001. More Han individuals (37.69%) than Mosuo individuals (23.35%) scored high security attachment. On the other hand, low attachment type has a significant higher percentage (29.65%) for the Mosuo than Han counterparts (11.05%). Results from *χ*^2^ test also show no significance in attachment to maternal mother among different ethnic groups (*χ*^2^ = 2.10, *p* > 0.05).

#### Ethnic Differences in Attachment Scores

**Figure 1:**
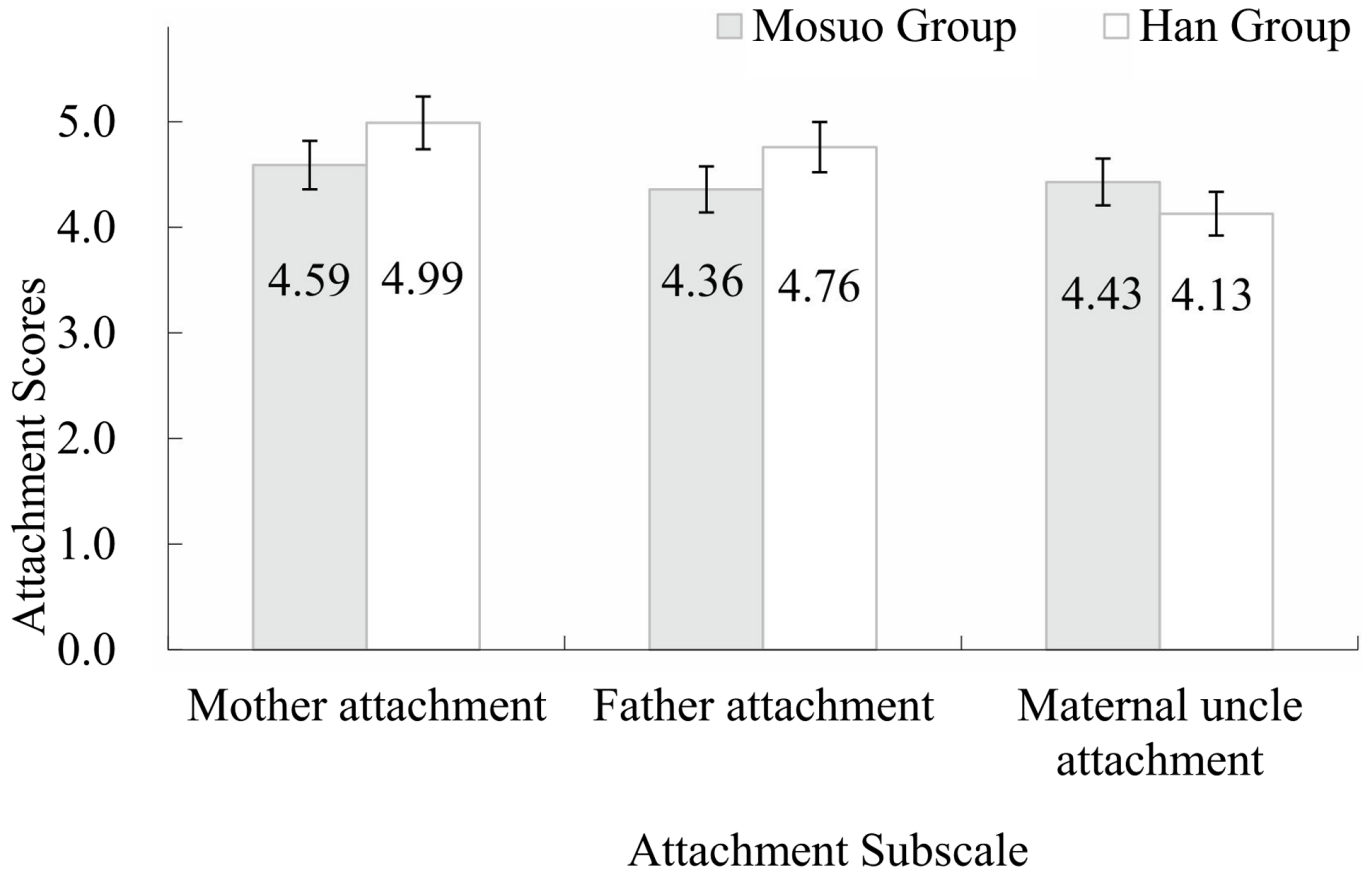
Average scores of the three attachment subscales between Mosuo Group and Han Group in Study 1. Error bars represent 95% CIs.

The scores of Mosuo participants and Han participants in the mother attachment subscale, father attachment subscale, and maternal uncle attachment subscale were calculated. A two (ethnic groups: Mosuo group vs. Han group) by three (attachment styles: mother attachment vs. father attachment vs. maternal uncle attachment) mixed-model ANOVA was conducted (see Figure 1). A main effect of ethnic groups was revealed, *F* (1,511) = 19.01, *p* < 0.001, *η*^2^ = 0.04. Mosuo group marked a significant lower score than Han groups. A main effect of attachment styles was also significant, *F* (2,1022) =169.55, *p* < 0.001, *η*^2^ = 0.25. Multiple comparison confirmed that attachment to mother was the strongest (with average score of 4.74 out of 5), second to which was father attachment (with average score of 4.52), and attachment to maternal uncle was the weakest among the three (with average score of 4.14). The interaction effect of ethnic group and the attachment type was also significant, *F* (2,1022) =21.11, *p* < 0.001, *η*^2^ = 0.04. Simple effect analysis demonstrated that Mosuo’s attachment to mother (*M ± SD* = 4.59 ± 0.87) significantly scored higher than father (*M ± SD* = 4.37 ± 0.84) or maternal uncle (*M ± SD* = 4.43 ± 0.79). Notably, no significant difference between attachment to father and maternal uncle was observed. Han participants had a similar pattern of significance between the score of attachment to mother, father, and maternal uncle. However, the Han group recorded higher score of both mother and father attachment than the Mosuo group. In contrast, Mosuo group displayed a significantly higher score of attachment to maternal uncle (*M ± SD* = 4.43 ± 0.79) over Han counterpart (*M ± SD* = 4.13 ± 0.86), suggesting that Mosuo subjects exhibited similar level of attachment to father and maternal uncle while Han displayed a stronger attachment to father.

## Study 2

As mentioned in Introduction, external driver factors, such as education, modernization, and tourism, have brought about significant changes in the Mosuo lifestyle in the last decade. In study 2, adults and juveniles are compared regarding response to the parent–child obligation relation, in order to reflecting the generation gaps between younger generation and older generation. The maternal uncle–nephew relationship and the father–child relationship are in sharp contrast in Mosuo culture, as revealed in Study 1. Study 2 compared both Han and Mosuo adult vs. juvenile groups reacting to one’s obligations as a father and a maternal uncle. This study hypothesized that, influenced by both the traditional Mosuo culture and the change of family structure, Mosuo adults may still favor their sisters’ children in assigning emotional and material resources (relatively speaking), Mosuo juvenile may show a similar pattern to their Han counterparts in their responses.

### Participants

The sample in Study 2 was grouped into adults and juveniles, with 107 Mosuo adults (61 males and 46 females, mean age = 28.27 ± 7.78 years) and 116 Han adults (58 males and 58 females, mean age = 29.74 ± 2.27 years). Meanwhile, 272 Mosuo juveniles in total were surveyed (124 males and 124 females and 24 members without clear information of gender, mean age = 17.34 ± 1.06 years). For the Han counterparts, 100 juveniles (47 males and 40 females, with 13 gender information missing, mean age = 16.92 ± 0.97 years) were recruited. Adults were recruited from Yong Ning village, Ninglang County, Yunnan province. Because all the participants were required to be literate and be able to communicate with researchers in Mandarin (the national language of China), some older villagers were excluded. The juveniles were students from the same local middle school. The study was approved by the Scientific Research Ethics Committee of Hangzhou Normal University. All adult participants were informed of the nature and content of the study and provided either written or oral consent. Similar to Study 1, Parents or teachers or other legal guardians were informed of the nature and content of the study and gave either oral or written consent to allow their minor children to participate, and the minors also gave their oral assent prior to participating in the study. All participants received a gift for completing the study.

### Materials and Procedure

A questionnaire was designed to measure how individuals assign rights and responsibilities with regard to father–child and maternal uncle–child relationship. Each situation (rights vs. responsibility) contains 12 specific vignettes, representing various common life scenarios that the participants may encounter.

The first set of scenario-based questionnaire was completed by adult participants, which mainly concerns the rights and responsibilities to son/daughter and maternal nephew/niece. It explored the right and responsibility assignment of Mosuo group and Han group, including adults comparing the responsibilities of one’s own children with nephews/nieces, and juveniles comparing father’s responsibilities with maternal uncle’s. Right and responsibility complement each other. For the purposes, right refers to one’s privileges granted by the society while responsibility refers to duties one needs to perform (usually to benefit the society). In this study, the adult participants were forced to make the decision regarding fulfilling his/her responsibility between X’s own child vs. his/her maternal nephew/niece in concrete cases. An example of the responsibility category is as follows:

X’s son/daughter and maternal nephew/niece were admitted by high school. Limited by 260 financial budget, X could only support one of them to go to high school. Who do you think X 261 would be more likely to support?

An example of the right category is as follows:

X is a well-known carpenter with great craftsmanship. Who do you think X would possibly pass his skill to?

The second set of scenarios was completed by juvenile participants, which contains questions on father and maternal uncle’s rights and responsibilities. The juvenile participants were similarly forced to make the decision between father vs. maternal uncle regarding their responsibility. An example of the responsibility is below:

X became the main breadwinner of the family when he grew up. Which elderly would X more likely to support?

An example of the right is below:

X bought many gifts with his first-month salary. Who do you think would be more likely to receive the more valuable gift?

In this scenario-based vignette study, participants were presented with a booklet containing specific scenarios with dilemmas and were asked to make a forced decision. Basic demographic information including age, ethnicity, gender was collected, and written informed consent was obtained from all participants.

### Results

#### The Adult Participants’ Forced Choices to the First Series Scenarios

Among the first set of scenarios, Scenario 1, Scenario 5, Scenario 6, and Scenario 9 are broken into responsibilities, and the other eight scenarios are broken into rights. The average counts of responses are recorded from adults’ forced choices, which show the attitudes regarding what the protagonists should choose in the context concerning rights and responsibilities between their own children and their sister’s children. A *χ*^2^ test was performed between Mosuo group and Han group responses. The results showed that adult participants’ choice between their son/daughter and maternal nephew/niece significantly differed between the Mosuo and the Han in both rights (*χ*^2^ = 55.32, *p* < 0.001) and responsibilities scenarios (*χ*^2^ = 58.47, *p* < 0.001).

In general, a similar percentage of Mosuo participants gave rights to their own children and their nephews/nieces. In contrast, significantly more Han participants chose to favor their own children. For example, in scenario 1, close to half (45%) of Mosuo participants chose to support their own children to study when financial resources are limited and this proportion reaches 90% in the Han group. In Scenario 10, 54% Mosuo participants chose maternal nephews/nieces to pass on the ancestral treasures, while only 3% Han counterparts made the same choice. In fact, in all scenarios, the Han participants were more likely to assign rights and responsibilities to their own children than nephews/nieces, sometimes significantly so.

**Figure 2:**
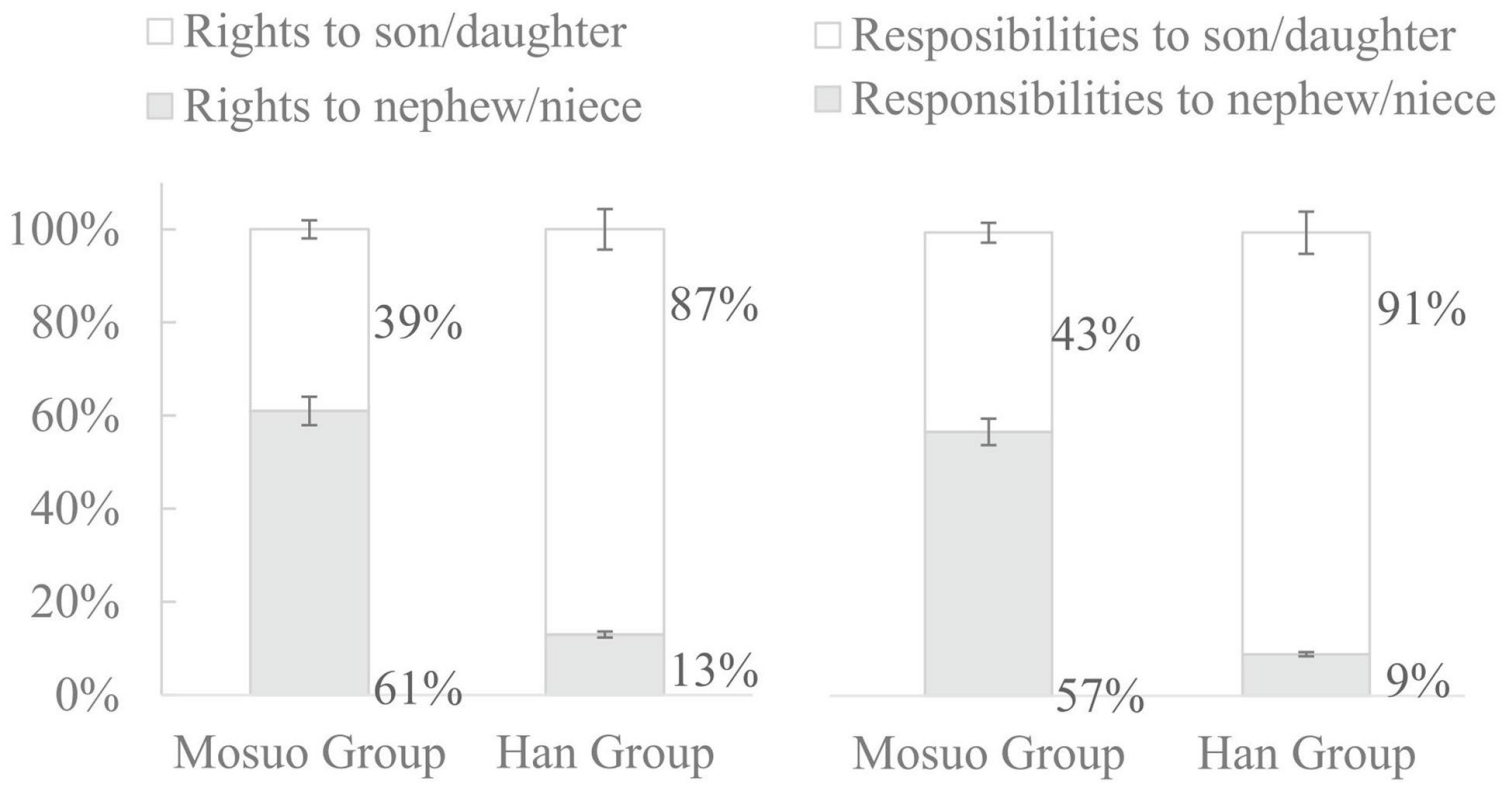
Adult participants’ average percentage of the choice on the rights and responsibilities in Study 2. Error bars represent 95% CIs.

The average percentage of choice favoring one’s nephew/niece on rights and responsibilities was calculated, respectively. A two (ethnic groups: Mosuo group vs. Han group) by two (obligation styles: rights vs. responsibilities) mixed-model ANOVA was conducted. A main effect of ethnic groups was revealed, *F* (1,221) = 177.28, *p* < 0.001, *η*^2^ = 0.44. Mosuo participants had a significant higher proportion choosing the option favoring maternal nephew/niece (0.59) than Han participants (0.11). A main effect of obligation styles was also significant, *F* (1,221) = 7.18, *p* < 0.05, *η*^2^ = 0.03, meaning the average proportion of individuals picking the choice favoring maternal nephew/niece (0.37) was significantly higher than that to the responsibility (0.33). The interaction between ethnic groups and rights and responsibilities was not significant, *F* (1,221) = 0.02, *p* > 0.05. The further ethnic differences on rights and responsibilities are shown in Figure 2.

#### The Juvenile Participants’ Forced Choices to the Second Series Scenarios

Among the second set of scenarios, the first four scenarios involved the rights for the father and maternal uncle, and the other eight scenarios involved their responsibilities for father and maternal uncle. The results of juvenile participants were analyzed similarly to those of adult participants. There is the general trend that most of juveniles in both ethnicities chose father to take on both rights and responsibilities. *χ*^2^ test showed that significant ethnic differences on responsibilities (*χ*^2^ = 5.00, *p* < 0.05), and the ethnic differences on rights were marginal significant (*χ*^2^ = 3.39, *p* = 0.07). In all eight scenarios concerning responsibilities, the proportion of Han group choosing father (83.88%) was higher than that of Mosuo group (74.86%).

**Figure 3:**
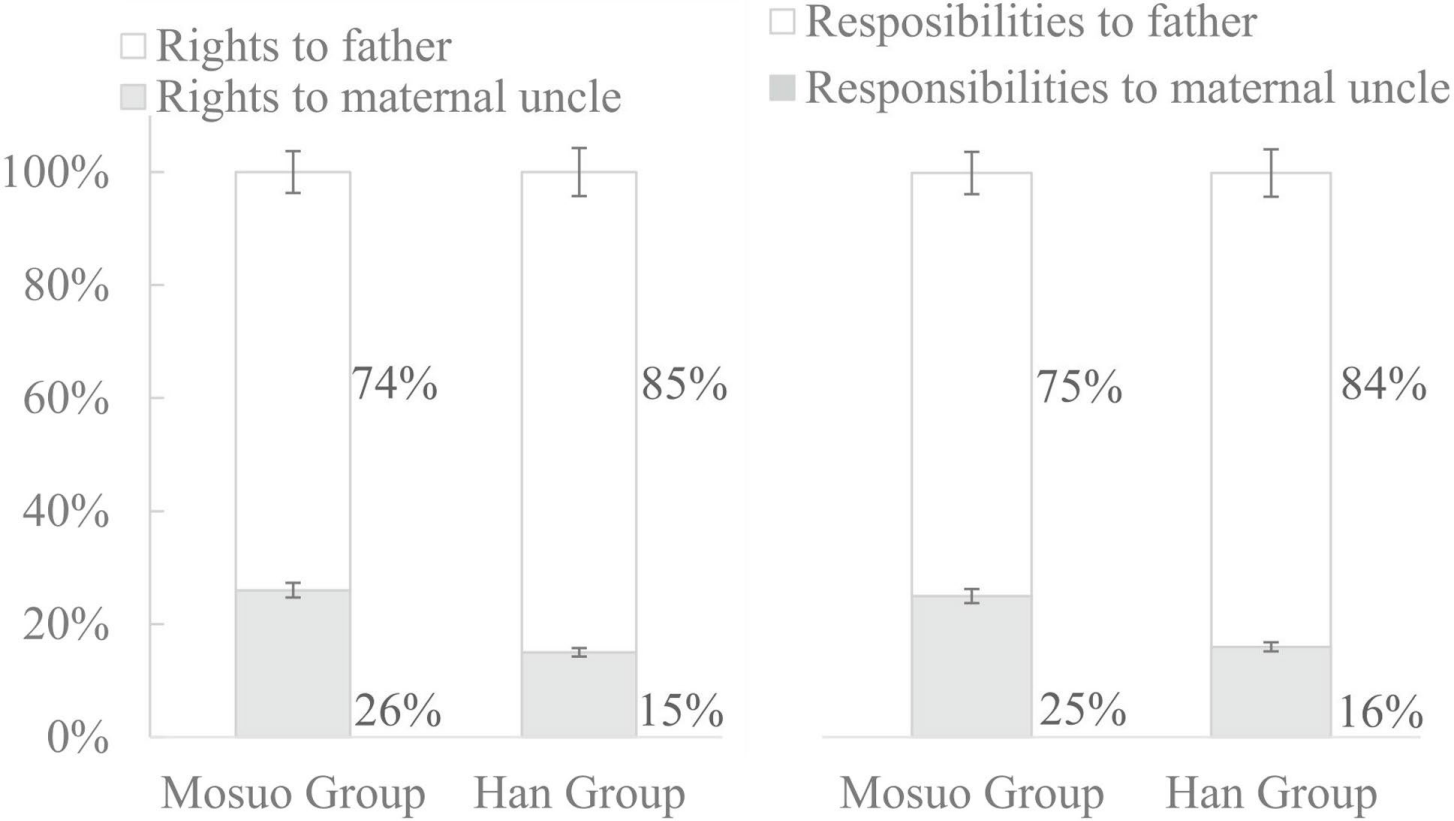
Juvenile participants’ average percentage of the choice on the rights and responsibilities in Study 2. Error bars represent 95% CIs.

The average percentage of choice favoring one’s father or maternal uncle on rights and responsibilities was calculated, respectively. A two (ethnic groups: Mosuo group vs. Han group) by two (obligation styles: rights vs. responsibilities) mixed-model ANOVA was conducted. A main effect of ethnic groups was revealed, *F* (1,370) = 12.49, *p* < 0.001, *η*^2^ = 0.03. The Mosuo participants had a significant higher proportion of picking choices favoring the maternal uncle (0.26) than their Han participants (0.15). The main effect of obligation styles [*F* (1,370) = 0.04, *p* > 0.05] and the interaction between ethnic groups and rights and responsibilities [*F* (1,370) = 0.55, *p* > 0.05] were not significant. The further ethnic differences on rights and responsibilities are shown in Figure 3.

## Discussion

The results support the hypothesis that there exist significant attachment style differences between the Mosuo and the Han participants. Specifically, in both groups attachment to mother exceeded the other two attachment styles. Several studies from different cultures support view of Bowlby (1969) that attachment and maternal behaviors are species characteristic adaptations (Ainsworth and Marvin, 1995; De Wolff and van IJzendoorn, 1997; Posada, 2013). The Mosuo showed no difference in levels of attachment with fathers and with maternal uncles, while the Han displayed a markedly stronger attachment with fathers than that with maternal uncles. Additionally, the Han group exhibited higher level of attachment for both parents than the Mosuo group. The Mosuo group, on the other hand, scored higher in attachment to maternal uncles than in attachment to fathers. There were no systematic sex differences in subjects’ attachment scores.

This study contributes to the growing literature of examining the cultural determinants in psychological and behavioral outcomes (Henrich et al., 2010). A series of studies have found that the cognition of kinship is affected not only by language (Zhang and Chen, 2004a), but also by individual experience (Zhang and He, 2004b; Xiao et al., 2010), cultural background, and circumstance (Zhang et al., 2013). The above results can be satisfactorily explained by the cultural contexts, in particular, family structure differences in two ethnic groups. In a Mosuo household, the role of the mother features prominently in the family from the child’s perspective, and the maternal uncle also enjoys high status as blood relative of the mother. A Mosuo family typically includes members bound by the ties of maternal kin. Grandmothers, maternal granduncles, mothers, mothers’ siblings, and children live in the same household (Cai, 2002; Shih, 2008). Mosuo children are raised by the maternal household, and children’s father is often excluded from the household. Maternal uncles play an important male role in the household. The Chinese old saying “the eagle holds the supreme power over the sky and the maternal uncle does the same but on the land” vividly describes the privilege and the honor a maternal uncle holds in a traditional matrilineal family. However, in a Han household, father, mother, and children are often the main members of the nuclear family. Maternal uncle is a close relative, but not the main member of the family. From this point of view, Han culture highlights the different roles between maternal uncle and father in a household, which is reflected by the difference of children’s attachment to them.

From an evolutionary perspective, if paternity certainty is low, it could be in a man’s best interest to invest in his sisters’ offspring, to whom his relatedness is assured, rather than to raise offspring to whom he might be unrelated (Trivers, 1972; Alexander, 1974; Greene, 1978; Gaulin and Schlegel, 1980; Anderson, 2006), and much empirical work has shown that paternity confidence is positively associated with the level of paternal investment (Gaulin and Schlegel, 1980; Marlowe, 1999; Anderson, 2006; Anderson et al., 2006; Huber and Breedlove, 2007). Among the Mosuo, it is possible that low paternity was induced by the specific ecological conditions and subsistence practices (or even social factors, such as increased sexual agency and partner choice), and men’s shifted investment toward their nephews/nieces may be viewed as an adaptive response in such environments. More recently, evolutionary anthropologists have specifically proposed that inherited wealth to sons relative to daughters play a role in predicting the kinship structure that is, matriliny and patriliny (Holden et al., 2003), which echoes the observation in the Mosuo society that wealth is often transferred to daughters rather than sons. Although these societal features are not the primarily focus for psychologists, they nonetheless contribute to the social–emotional interaction between maternal uncles and nephews/nieces, therefore affecting the attachment levels as measured in our studies.

The findings in the study also suggest the impact of modern mainstream patriarchal culture on Mosuo culture. From 1983, the Chinese Government encouraged the Mosuo people to develop tourism. People from many different cultural backgrounds visited the Mosuo and tourism substantially undermined traditional Mosuo culture. One of the results is the interethnic marriage. Marriage with outsiders, especially with the Han people, has changed the size and the structure of the modern Mosuo family and the relationships among family members. Noticeably, the size of the Mosuo family is becoming smaller and the Mosuo fathers increasingly take financial responsibility and become involved in educating their own children. Also, with the increased interaction between fathers with their children, father–child relationship has been greatly improved and becoming tighter. Juvenile participants in the current study are middle school students, most of whom still live in traditional matrilineal “big family” and only a small proportion is living in the nuclear family with only parents and siblings. Yet even when the fathers do not physically live with their children, they would frequently visit their own children, and as a result, the children’s attachment to father is becoming stronger. At the same time, the children also maintain very close contact with their maternal uncles. Therefore, the results show that children have similar types of relationships with their father and maternal uncle with regard to attachment, which partly explains the similar attachment scores for fathers and maternal uncles for the Mosuo in Study 1. Additionally, it is possible that the increased paternity certainty among the Mosuo in recent years as a result of monogamous relationships (Xiao et al., 2015) triggers an adaptive response for Mosuo men to invest more in their own children, therefore strengthening the father–child relationship in the family.

In the Han culture, influenced by the value that “men work outside and women work inside,” the mother takes the role of children’s daily career and communicate constantly with them (Levy, 2013). In contrast, father–child communication and interaction is relatively less. Therefore, it makes sense that children develop stronger trust and attachment toward their mother than their father. The maternal uncle is usually perceived to be an “outsider” of the nuclear family, which makes their daily contact with the children minimal, and therefore, the lowest attachment score for maternal uncles is observed among the Han.

Some additional insight could be gained from a deeper look at kin terms. Notably, in Mosuo language there is no linguistic distinction among biological mother, foster mother, stepmother, and even all of mother’s sisters. They are collectively referred to as “Ami,” and the word “Amizhi” and “Amiji” are only used to distinguish age rather than closeness (Zhan et al., 2006; Xiao et al., 2010). Research shows that “self-reference” is one of the boundary conditions of Retrieval-Induced Forgetting (RIF). In the Han Chinese culture, RIF was observed under the conditions of self-reference and maternal reference; while in Mosuo culture, mother’s sisters who are integrated in the self-concept of the Mosuo people are also important to such individuals (Wang et al., 2019). To some extent, Mosuo people consider their mother and mother’s sisters as equally important, which may “dilute” the attachment devoted to the biological mother. In the Han culture, one’s biological mother and the mothers’ sisters have very distinct linguistic labels and are perceived quite differently regarding their closeness to the ego, which might explain why Mosuo attachment to mother is less strong in comparison with Han. Similarly, in the Chinese language (standard mandarin), the kin term “jiujiu” (maternal uncle) refers to mother’s brother, indicating a kind of indirect blood relationship. In contrast, in the Mosuo language both maternal uncle and biological father are referred to as “awu” (Peng, 1997), suggesting that the importance of maternal uncle in Mosuo culture is comparable to that of the father. What is perhaps more interesting is that calling father “awu” shows respect to the biological father and better contributes to the harmony of matrilineal family (Zhou, 2010). The results in Study 1 thus reflect the characteristics of specific family relationship of the two populations. Maternal uncle–nephew/niece relationship resembles that of father–son/daughter, and nicely manifests the uniqueness of Mosuo social and family structure. Interestingly, attachment to a child’s father and his/her maternal uncle differs from Han culture.

Results from scenario-based questionnaires in Study 2 show that the Mosuo adults were more likely to take on rights and responsibilities for their maternal nephew/niece than their own children than their Han counterparts. For juveniles from both ethnicities, the majority chose father to take on rights and responsibilities, though this percentage is lower for the Mosuo.

In Mosuo family, the maternal uncle shares the responsibility of raising and educating the child together with the mother and as a result, he enjoys the right to be respected and given proper burial after death by his nephew/niece. In contrast, Mosuo children received rather limited financial and emotional input from father (this is especially true in the past), thereby are less responsible for their biological father when they grow up. In recent years, due to contact with and influence from the Han culture, father’s role in Mosuo family becomes increasingly important, as reflected in father’s input in raising and educating their children in the family. Therefore, attachment to father among younger generation becomes stronger than earlier times, as suggested by results in Study 2. Interestingly, the majority of participants in both ethnicities chose father to take on more responsibility than the maternal uncle, though the percentage of the Mosuo juveniles making the choice was smaller than the Han group. Among the Han, adults would easily prioritize their own child over nephew/niece. However, it was not that easy to make the choice for the Mosuo adult participants. During the field research, Mosuo people took much more time and hesitation before they made the choice between son/daughter and nephew/niece, suggesting maternal uncle’s changing role between different generations.

The typical Mosuo family nowadays mixes traditional and modern values, and the traditional aspect of their culture is met with great challenges by the process of material modernization and cultural homogenization (Xu, 2006). Most notably, the changing roles of maternal uncle and biological father in the family incurs severe conflicts. In general, mother and maternal uncle still hold more power and exert more influence on the children in the household yet the importance of the father is gradually increasing. With the increasing number of families adopting monogamy, father and mother start to share financial and educational responsibilities of their children. The conflicts of rights/responsibilities between the maternal uncle and father are very much ongoing and occurs in both adults and juveniles as the results have shown, but non-trivial generational differences are observed: juveniles are affected by the Han culture more than adults.

Today, Mosuo male adults are the main source of family income and are obliged to financially support their maternal uncle as he reaches seniority. With the influence of monogamous Han culture, they have to at the same time support their own biological father. As fathers and maternal uncles themselves, the modern Mosuo adults are paying more attention to their own children and at the same time still connecting tightly to their maternal nephews and nieces. Therefore, in many cases, the modern Mosuo adults treat their own children and their maternal nephews and nieces rather similarly. It is worth re-emphasizing the point that modern commercial economy and the culture of monogamy probably have a bigger effect on Mosuo juveniles than adults, as the increasing importance of father in a family likely reshapes the father’s image in children’s mind. As more Mosuo individuals adopt the nuclear family structure, their traditional matrilineal family and the unique parenting mode are likely to be disappear in the near future.

## Conclusion

This paper presents evidence from two studies showing that the matrilineal Mosuo and the patrilineal Han exhibit markedly different levels of attachment toward maternal uncle: the Mosuo, due to their unique marriage and family structure, are much more likely to be closely attached to their maternal uncle compare to the Han where the role of maternal uncle in the family is minimal. Additionally, the results show that Mosuo adults are much more likely to attribute rights and responsibilities to maternal uncle than their Han counterparts in a series of vignette scenarios, whereas much of a difference was not observed in Mosuo juveniles, reflecting the changing roles of the father/maternal uncle of the Mosuo with the cultural influence of the Han majority.

## Data Availability Statement

The datasets presented in this study can be found in online repositories. The names of the repository/repositories and accession number(s) can be found at: https://osf.io/qdkga.

## Ethics Statement

The studies involving human participants were reviewed and approved by the Scientific Research Ethics Committee of Hangzhou Normal University. Written informed consent to participate in this study was provided by the participants’ legal guardian/next of kin.

## Author Contributions

EX and JZ conceptualized the idea and designed the study. EX and JJ analyzed data and drafted the manuscript. ZH guided and supported by critically reviewing the manuscript. All authors contributed to the article and approved the submitted version.

## Funding

This work was supported by the Provincial Key Cultivation Project of Hangzhou Normal University under Grant 20JYXK013.

## Conflict of Interest

The authors declare that the research was conducted in the absence of any commercial or financial relationships that could be construed as a potential conflict of interest.

## Publisher’s Note

All claims expressed in this article are solely those of the authors and do not necessarily represent those of their affiliated organizations, or those of the publisher, the editors and the reviewers. Any product that may be evaluated in this article, or claim that may be made by its manufacturer, is not guaranteed or endorsed by the publisher.
